# Measuring distance through dense weighted networks: The case of hospital-associated pathogens

**DOI:** 10.1371/journal.pcbi.1005622

**Published:** 2017-08-03

**Authors:** Tjibbe Donker, Timo Smieszek, Katherine L. Henderson, Alan P. Johnson, A. Sarah Walker, Julie V. Robotham

**Affiliations:** 1 The National Institute for Health Research (NIHR) Health Protection Research Unit in Healthcare Associated Infections and Antimicrobial Resistance at University of Oxford in partnership with Public Health England, Oxford, United Kingdom; 2 Nuffield Department of Medicine, University of Oxford, Oxford, United Kingdom; 3 National Infection Service, Public Health England, Colindale, London, United Kingdom; 4 MRC Centre for Outbreak Analysis and Modelling, Department of Infectious Disease Epidemiology, School of Public Health, Imperial College London, London, United Kingdom; 5 NIHR Biomedical Research Centre, Oxford, Oxford, United Kingdom; Johns Hopkins Bloomberg School of Public Health, UNITED STATES

## Abstract

Hospital networks, formed by patients visiting multiple hospitals, affect the spread of hospital-associated infections, resulting in differences in risks for hospitals depending on their network position. These networks are increasingly used to inform strategies to prevent and control the spread of hospital-associated pathogens. However, many studies only consider patients that are received directly from the initial hospital, without considering the effect of indirect trajectories through the network. We determine the optimal way to measure the distance between hospitals within the network, by reconstructing the English hospital network based on shared patients in 2014–2015, and simulating the spread of a hospital-associated pathogen between hospitals, taking into consideration that each intermediate hospital conveys a delay in the further spread of the pathogen. While the risk of transferring a hospital-associated pathogen between directly neighbouring hospitals is a direct reflection of the number of shared patients, the distance between two hospitals far-away in the network is determined largely by the number of intermediate hospitals in the network. Because the network is dense, most long distance transmission chains in fact involve only few intermediate steps, spreading along the many weak links. The dense connectivity of hospital networks, together with a strong regional structure, causes hospital-associated pathogens to spread from the initial outbreak in a two-step process: first, the directly surrounding hospitals are affected through the strong connections, second all other hospitals receive introductions through the multitude of weaker links. Although the strong connections matter for local spread, weak links in the network can offer ideal routes for hospital-associated pathogens to travel further faster. This hold important implications for infection prevention and control efforts: if a local outbreak is not controlled in time, colonised patients will appear in other regions, irrespective of the distance to the initial outbreak, making import screening ever more difficult.

## Author summary

Shared patients can spread hospital-associated pathogens between hospitals, together forming a large network in which all hospitals are connected. We set out to measure the distance between hospitals in such a network, best reflecting the risk of a hospital-associated pathogen spreading from one to the other. The central problem is that this risk may not be a directly reflected by the weight of the direct connections between hospitals, because the pathogen could arrive through a longer indirect route, first causing a problem in an intermediate hospital. We determined the optimal balance between connection weights and path length, by testing different weighting factors between them against simulated spread of a pathogen. We found that while strong connections are important risk factor for a hospital’s direct neighbours, weak connections offer ideal indirect routes for hospital-associated pathogens to travel further faster. These routes should not be underestimated when designing control strategies.

## Introduction

A growing body of literature shows the influence of contact networks on the spread of infectious diseases. This extends from individual hosts [1–5] to entire countries [6–10] or organisations, such a healthcare institutions for pathogens that are predominantly transmitted through hospital contacts [11–14]. The focus on contact networks, between various entities, reflects the importance of social structure on the successful spread of a pathogen, and the fact that the risk of contracting an infectious disease depends not only on the properties of the host or the pathogen [15]. Each contact between an entity may potentially serve as a transmission route, aiding pathogen spread.

Traditionally, individuals in a person-based network were considered to be connected simply when there was any contact between them over the study period, resulting in an unweighted (binary) network; the characteristics of a contact, such as its length or intensity [16–18], were not taken into account. However, advances in techniques to determine the level of contact between individuals, such as wearable sensors that measure face-to-face contact [4, 19–25], have resulted in more weighted networks becoming available. Like these recent person-to-person networks, hospital networks comprise weighted connections between institutions, as the number of shared patients (sometimes compensated for their length of stay) can be used to estimate the extent of contact.

However, some differences between these networks should be considered. Person-to-person contact is almost by definition symmetrical: if person A was in close contact with person B, the reverse should also be true [22, 23]. In contrast, hospital networks are nearly always asymmetrical, because the number of patients moving from hospital A to B is not necessarily the same as the number of patients moving from B to A. Furthermore, the geographical position of the hospital nodes is fixed and usually known, making it straightforward to compare network and geographical distances.

Previous studies have shown how infection prevention and control (IPC) efforts against hospital-associated pathogens in general, and bacteria showing antimicrobial resistance (AMR) in particular, should be coordinated according to the structure of the hospital network [26]. Correct estimates of the network distance between hospitals are therefore important for risk assessments during outbreaks of hospital-associated pathogens. However, this is complicated by the properties of these hospital networks. While the distance between two individuals in an unweighted network is simply the minimum number of steps needed to reach one from the other [27], the distance through a dense weighted network is less straightforward, because of the interplay between the number of intermediate steps and the weight of the contact [28]. A direct weak link may, for instance, constitute a longer distance than a route through many intermediate nodes, but with stronger links.

We here estimate the distances between hospitals in the English hospital network, while considering the spread of AMR as an example. AMR poses a significant risk to hospitalised patients, and effective IPC is paramount [29]. We simulate the spread of hospital-associated antibiotic-resistant pathogens with various transmission characteristics, to determine the optimal balance between the number of intermediate steps and the weight of the connections to measure the connectivity between hospitals in England. We compare the network distance to geographical distances to determine the potential differences between them. The resulting distance metric can inform IPC strategies after observed outbreaks.

## Materials and methods

### Hospital network

We used data from the English National Health Service (NHS) Hospital Episode Statistics (HES) for financial years 2013/14 and 2014/15 to construct a network of hospital organisations (trusts) in England based on patient movements. The NHS-HES includes all inpatient admissions to hospital trusts in England, based on provider episodes. We included all acute care hospital trusts, including specialist hospitals (such as oncological), and joined all consultant episodes per admission (called a ‘spell’ in NHS-HES, the time between admission and discharge in the same hospital).

We sorted the spells per patient by admission date, and for each admission during 2014/15 determined if the previous discharge (if any) happened within the 365 days prior to the admission. For each pair of discharge and subsequent admission event we tallied the two hospitals involved in this single patient movement (*m*_*ij*_). Information from all spells during 2014/15 taken together thus form a contact matrix, *M*_*ij*_, of patient movements between hospitals *i* and *j*. This contact matrix therefore included both direct (i.e. patient transfers between hospitals) and indirect (i.e. with a stay at home in between) patient movements.

Each node in the network represented a single hospital trust and the edges represented the number of patients who moved from one hospital trust to the other. The network was weighted (number of patients moving) and directional (in any pair of hospital trusts, the numbers of patients moving from and to each hospital could differ). Based on the community structure of the measured hospital network, we divided hospitals into referral regions. The community assignment with maximum modularity was determined using a standard community structure detection algorithm [30].

### Simulations

We simulated the spread of a hospital-associated pathogen through the hospital network using a stochastic, discrete-time model with time steps (ΔT) of 7 day, much in the same way as used by Ciccolini et al. [31] and Van Bunnik et al [32]. In the simplest version of the model, we assume that each hospital is either “susceptible” (pathogen not present) or “infected” (pathogen present), and infected hospitals have a fixed prevalence, which determines the proportion of discharged patients that are colonised with a hospital-associated pathogen. The probability of a hospital getting infected depends on the state of the other hospitals, the assumed prevalence, and the patient movements between them. The probability of getting infected in the initial model can be described as:

$$\begin{array}{c} P_{i}^{S\to I}=1-\prod_{j:H_{i}\in I}^{N_{H}}\;{\left(1-\gamma \right)}^{C_{I}M_{ij}\Delta T} \end{array}$$ (1)

where *H*_*j*_ denotes each hospital, whose introductions to hospital *j* are summed if they are part of the set of infected hospitals, *I*. *γ* is the between-hospital transmission parameter equal, to the probability that an imported colonised individual successfully spreads the pathogen. *C*_*I*_ is the attained prevalence after infection of the hospital. *M*_*ij*_ denotes the patient movements between hospitals *i* and *j*, as measured in the NHS-HES data. We chose parameter values such that about half of the hospitals were affected after one year, using an equilibrium prevalence (*C*_*I*_) of 20%. Table 1 lists the parameters used for the main analysis, as well the parameters values used to test the sensitivity of parameter choices.

**Table 1:** Model paramters.

Parameter	Description	Value (sensitivity analysis)	Results sensitivity analysis
*γ*	Chance of successful introduction (per colonised patients), using no within-hospital delay	5 × 10^−5^ (10^−3^, 10^−4^, 2 × 10^−5^, 10^−6^)	S2 Fig
*γ*	Chance of successful introduction (per colonised patients), using SIS within-hospital model	10^−3^ (10^−2^, 2 × 10^−3^, 5 × 10^−4^, 2 × 10^−4^)	S3 Fig
*β*	Within-hospital transmission parameter	0.1(1.0)	S4 Fig
*δ*	Within-hospital removal rate of colonised individuals	0.08 with *β* = 0.1 (0.01, 0.05, 0.09) and 0.8 with *β* = 1.0 (0.1, 0.5, 0.9)	Fig 4 and S4 Fig
*R*_0_	Basic reproduction number	1.25 [*β* = 0.1, *δ* = 0.08] (1.11 [*β* = 0.1, *δ* = 0.09], 2.0 [*β* = 0.1, *δ* = 0.05], 10.0 [*β* = 0.1, *δ* = 0.01] and 1.11 [*β* = 1.0, *δ* = 0.9], 1.25 [*β* = 1.0, *δ* = 0.5], 2.0 [*β* = 1.0, *δ* = 0.5], 10.0 [*β* = 1.0, *δ* = 0.1])	Fig 4 and S4 Fig
*I*(0)	Starting prevalence (% colonised)	0.1%	-

Because the assumed binary infection state of the hospitals is a fairly crude approximation of the within-hospital dynamics of hospital-associated pathogens (since the infectivity of a hospital is likely to increase after the initial successful introduction), we expanded the model to include the increasing prevalence within the hospital. We modelled this using a standard SIS model; with transmission rate *β* and removal rate *δ*, the infected class is described as

$$\begin{array}{c} \frac{dI}{dt}=\beta SI-\delta I. \end{array}$$ (2)

As the susceptible class is simply the rest of the total population, using *S* = 1 − *I*, the within-hospital epidemic curve can be derived as follows:

$$\begin{array}{c} I\left(t\right)=\frac{I(\infty )}{\left(1-\left(1-\frac{I(\infty )}{I(0)}\right)\right)e^{-(\beta -\delta )t}} \end{array}$$ (3)

where *I*(∞) denotes the equilibrium prevalence given by $I\left(\infty \right)=1-\frac{1}{R_{0}}$, with $R_{0}=\frac{\beta }{\delta }$, and *I*(0) the starting prevalence at introduction, set at 0.001 (i.e. one patient among a thousand admitted). This function can then be used to calculate the within-hospital prevalence at each time point,

$$\begin{array}{c} P_{i}^{S\to I(0)}\left(t\right)=1-\prod_{j:H_{i}\in I}^{N_{H}}\;{\left(1-\gamma \right)}^{I{\left(t\right)}_{i}M_{ij}\Delta T} \end{array}$$ (4)

The parameter values were chosen again such that approximately half the hospitals became affected after one year, together with a within-hospital *R*_0_ = 1.25, reflecting *R*_0_ values often used for studying the spread of AMR in hospital settings [33], resulting in an equilibrium prevalence of 20%.

We used each hospital in turn as the initial starting point of the outbreak, and repeated the simulation 100 times per starting hospital, resulting in 15200 iterations of the model, which delivers stable estimates (see S1 Fig). We tracked the time of infection per hospital, and estimated the mean time to infection for each pair of starting and receiving hospitals.

### Network distance, shortest path

**Fig 1:**
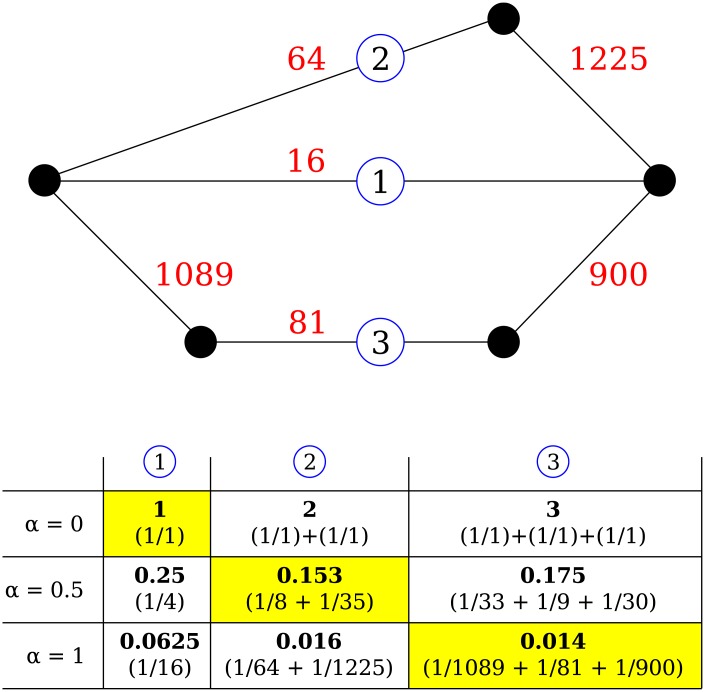
Schematic representation of measuring the network distance depending on the scaling parameter *α*. Dots represent hospitals, red numbers show the number of patients shared between them. Route 1 is a direct route between the left and right hospital through a weak connection, while the other routes are indirect, through a mixture of strong and weak connections. The table shows the calculated distances for each route for three values of a (0.0, 0.5, and 1.0); Each route in this example can be the shortest (in yellow) depending on the exact value of *α*.

In order to determine the shortest paths between hospitals, we applied a metric proposed by Opsahl et al [28], that allows weighting between the number of steps and edge weight (Fig 1). The shortest path through the weighted network, *d*^*wα*^ between node *i* and *j* is

$$\begin{array}{c} d^{w\alpha }=\text{min}\left(\frac{1}{M_{ih}^{\alpha }}+\cdots +\frac{1}{M_{hj}^{\alpha }}\right) \end{array}$$ (5)

If *α* is set to 0, the shortest path is completely determined by the number of steps, as each existing connection has value 1, irrespective of the weight of the connection. Conversely, if *α* is set to 1, the shortest path is fully determined by the weight of the connections (i.e. the number of shared patients). Technically, *α* can be set to above one, where the shortest path is skewed towards the strongest links, but we considered only values within the range of 0 to 1.

### Centrality

To determine the risk posed by an outbreak in any of the other hospitals, we calculated the centrality of each hospital. We considered three centrality metrics: degree, closeness, and betweenness centrality. Closeness centrality is given by the inverse of the distances to all hospitals, as defined by the shortest paths

$$\begin{array}{c} C_{C}^{w\alpha }\left(i\right)=\sum_{j}^{N_{H}}\;\frac{1}{d_{w\alpha }\left(i,j\right)} \end{array}$$ (6)

while the betweenness centrality is defined as the proportion of all of these shortest paths that pass through node i,

$$\begin{array}{c} C_{B}^{w\alpha }\left(i\right)=\frac{g_{jk}^{w\alpha }\left(i\right)}{g_{jk}^{w\alpha }} \end{array}$$ (7),

where $g_{jk}^{w\alpha }\left(i\right)$ is the number of shortest paths passing through hospital *i*, and $g_{jk}^{w\alpha }$ is the total number of shortest paths between all hospitals (excluding hospital *i*: (*N* − 1)(*N* − 2)). We used the degree centrality defined as the sum over all connection weights, adjusted for the importance of steps by *α*

$$\begin{array}{c} C_{D-in}^{w\alpha }\left(i\right)=\sum_{j}^{N_{H}}\;M_{ji}^{\alpha } \end{array}$$ (8)

To determine the optimal balance between the number of intermediate hospitals and the strength of the connections to accurately reflect the spread of infectious pathogens through the hospital network, we calculated the shortest paths and centralities for various values of *α* and determined their correlation with the simulation results. We used Kendall’s tau (*τ*) to measure the correlation between the mean simulated time to infection and the distances and centrality metrics, as it gives the most intuitive interpretation compared to other measures of rank correlation (*τ* gives the proportion of randomly chosen pairs that are concordant) [34].

### Removing weak links

To investigate the influence of weak links (i.e. connections between hospitals exchanging few patients) that could theoretically be removed most easily, we repeated the analysis with altered hospital networks. For each alteration, we removed the links with a weight under a certain threshold, calculated the shortest paths between hospitals for various values of *α*, and repeated the simulations for the scenario using a binary hospital infection state (Eq 1) and the scenario using the SIS within-hospital model (Eqs 2 and 4).

## Results

### Network properties

**Fig 2:**
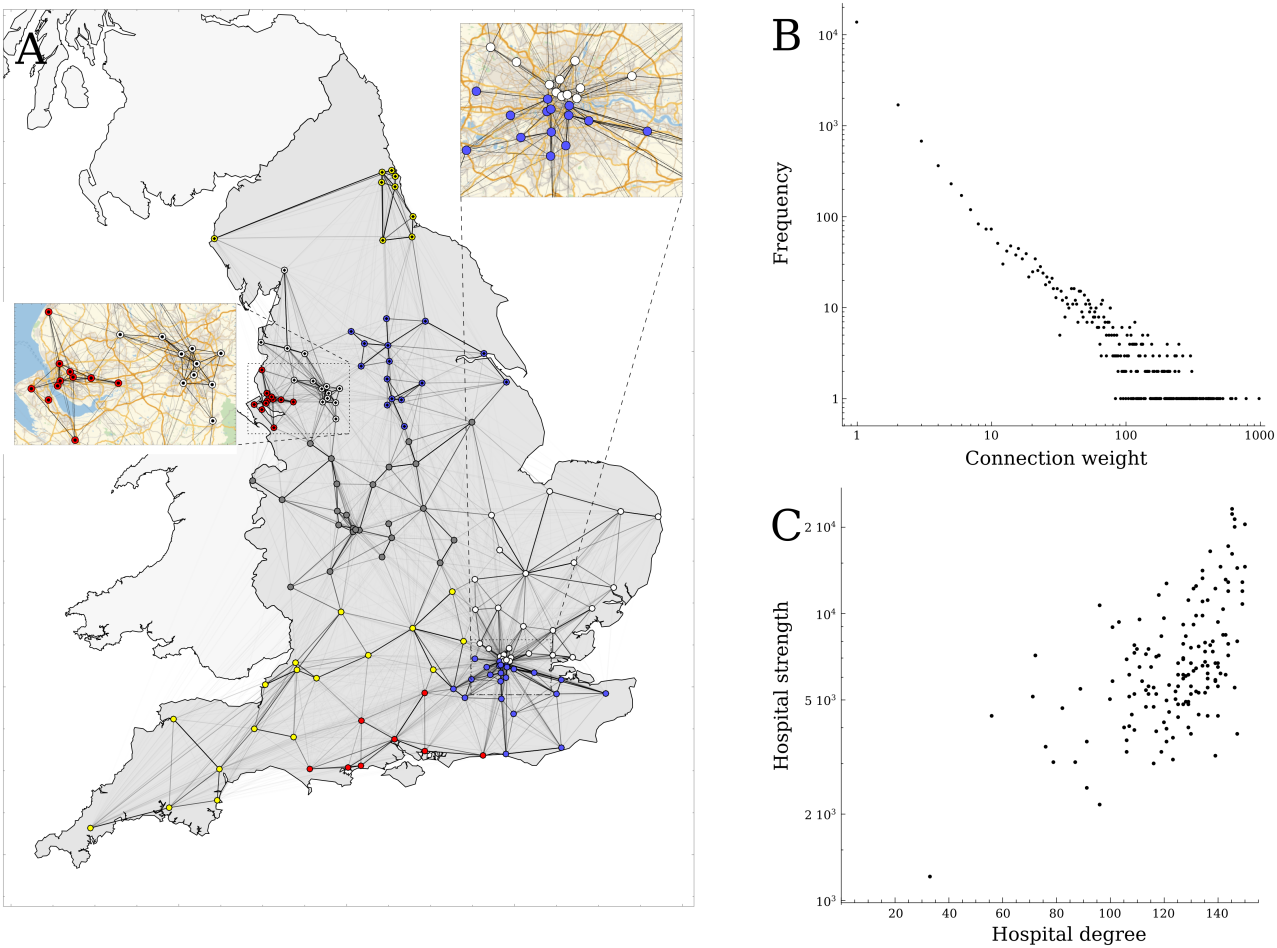
The patient movement network in England. A) Strong connections (darker lines) between hospitals (circles) are regionally bound, and form a strong community structure (circle colours). B) The distribution of connection weights shows that most connections between hospitals are weak (i.e. few shared patient between hospitals). C) The degree of a hospital (the number of hospitals it is connected to by shared patients) is related to the total number of patients it exchanges with other hospitals (hospital strength).

The hospital network consisted of 152 acute hospital trusts (Fig 2A) that admitted 4,091,977 patients with a total of 16,643,460 admissions in financial year 2014/2015. On average each hospital exchanged 7,481 (IQR: 4583–9244) patients with 124 (IQR: 114–137) other hospitals, resulting in 18,847 unique hospital to hospital connections made by individual patients, comprising 82% of all possible links. The weight of the connections was highly skewed (Fig 2B), with 90% of the connections based on movements of 50 patients or fewer, comprising 10% of the total number of patient exchanges. Although highly connected hospitals (i.e. those sharing patients with many other hospitals) shared more patients (Fig 2C), the mean number of patients exchanged per connection was not higher for the well-connected hospitals.

### Simulations

**Fig 3:**
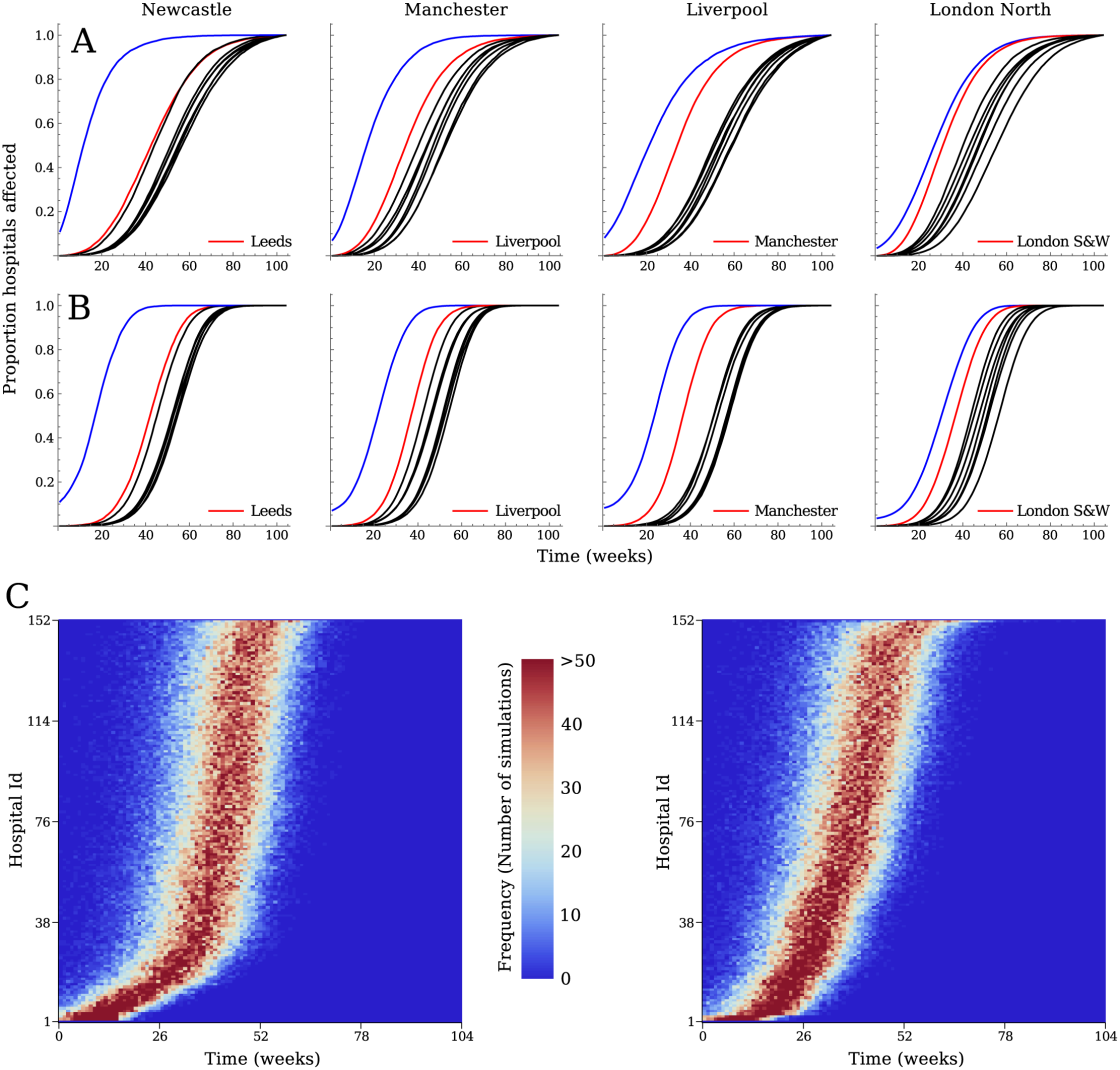
Results of the stochastic simulations, showing the mean proportion of affected hospitals over time, per referral region. A) shows the results for the model without delay, B) the results for the model with the SIS within-hospital model. The region of the initially affected hospital (Blue, each column corresponding to a different initial outbreak hospital as labelled) was always the first to be affected, while the timing of the other regions becoming infected depended on the starting point, with often a close by region (Red) affected next. C) The resulting distribution of time to infection from 1000 simulations starting at two randomly selected hospitals; hospitals are ordered along the vertical axis according the mean time to infection.

The simulated spread of a pathogen through the hospital network showed a fast increase in the proportion of hospitals within the referral region of the initially affected hospital where the pathogen was present, followed by the other regions after some time (Fig 3). The order in which the pathogen reached hospitals in other regions depended on the position (region) of the initially infected hospital. For some, the hospitals in one of the neighbouring regions were clearly the next ones to be affected (e.g. Liverpool after Manchester and vice versa), while for others (e.g. Newcastle) there was no discernible order of regions, and they were all affected at more or less the same time. The difference in time for the pathogen to reach the other hospitals in the starting region and hospitals in the other regions was shortest when London hospitals were the first affected.

When including a delay between the time a hospital became infected and the time it starts to spread the pathogen to its neighbouring hospitals, the difference in time to infection between regions became slightly bigger. In particular, the time from the initial region becoming infected to the other regions becoming infected increased, indicating that spread within the regions depended mostly on the direct connections with the initial hospital, while other regions were more often reached by indirect routes.

### Network distance

**Fig 4:**
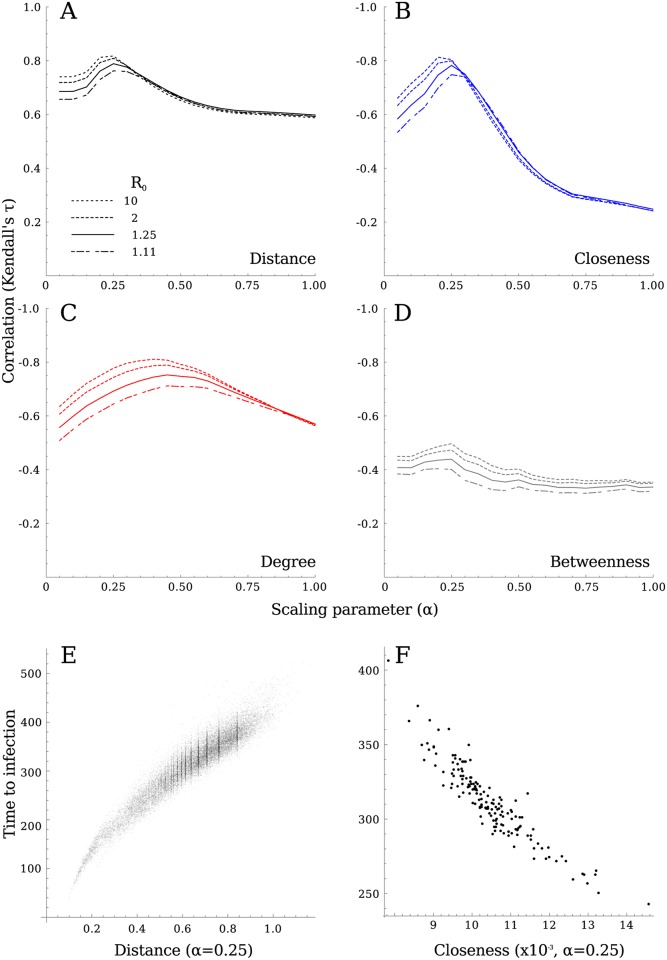
Determining the appropriate weighting between number of steps (*α* = 0) and the edge weight (*α* = 1) to measure the distance through the hospital network. A) The correlation between network distance and time to infection in the stochastic simulations shows a peak around *α* = 0.25. The exact value does not strongly depend on the modelled *R*_0_, or other model parameters (see S2–S4 Figs for further sensitivity analyses), with longer delays and higher introduction probabilities only slightly shifting the optimum to lower *α*. The mean time to infection, reflecting the risk of a hospital getting infected, relates to the hospital’s centrality. B) Although closeness showed the best correlation (at *α* = 0.30), C) degree centrality performs only marginally less well (at *α* = 0.50 − 0.60), while D) betweenness showed a poor correlation, with no clear optimal value for *α*. E) The time to infection as a function of shortest path length between all hospitals using *α* = 0.25, is strongly correlated with network distance. F) The correlation between the mean time to infection and closeness for each hospital.

The shortest path distance based purely on the connections weights (*α* = 1) correlated with the simulated time it took the infection to spread between any two hospitals (Fig 4A). However, incorporating the number of intermediate hospitals in the distance metric, by scaling factor *α*, increased the association with time to infection (Fig 4A). This association was maximal around *α* = 0.25, in particular for simulations with higher within-hospital *R*_0_ values. The shortest route between two hospitals therefore tended to have fewer steps (intermediate hospitals) instead of more exchanged patients. This implies that the route of transmission between two hospitals does not necessarily follow the connections between hospitals sharing many patients. The optimal value for *α* did not depend on the parameter choices in our model (S2–S4 Figs), and although some parameter combinations did show weaker or stronger correlations, the position of the maximal correlation only slightly altered.

**Fig 5:**
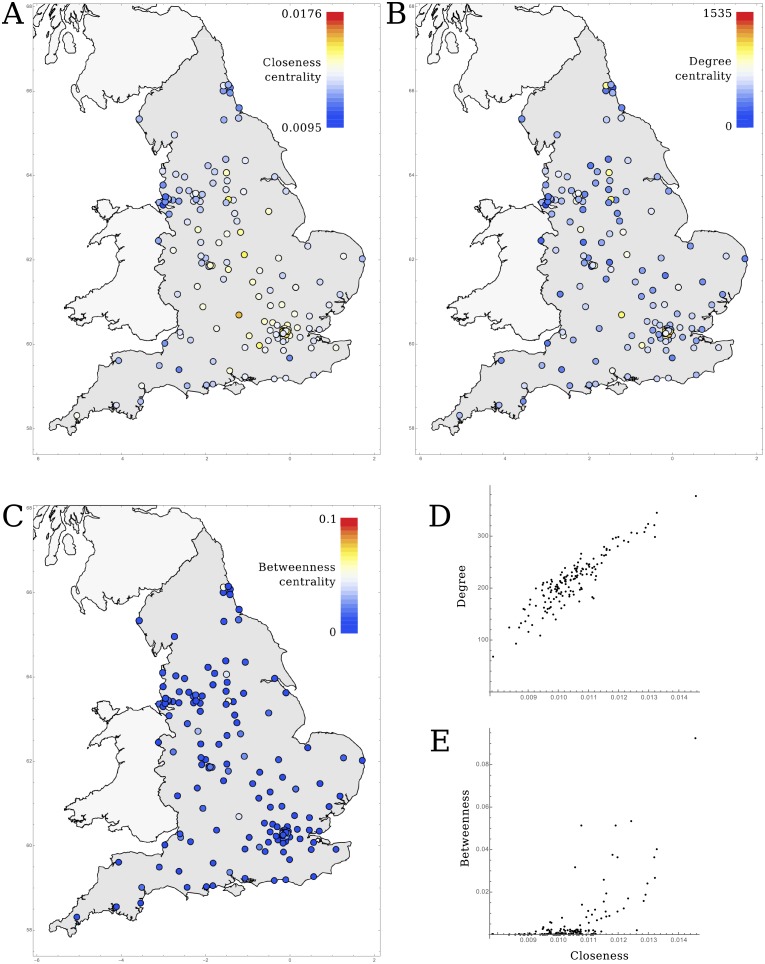
The distribution of centrality of hospitals in England, showing A) closeness centrality (at *α* = 0.25), B) degree centrality (at *α* = 0.55), and C) betweenness centrality (at *α* = 0.25). D&E) show the correlation between closeness and degree or betweenness, respectively. Closeness and degree centrality are strongly correlated, while only few hospitals with high closeness showed high betweenness.

If we consider the mean time to infection from all other hospitals, which translates to the general risk these hospitals pose to the focal hospital, this correlated well with closeness centrality (Fig 4B) at values of *α* around 0.2–0.3, again preferentially incorporating the direct weak connections over indirect strong routes. The –local– degree centrality (Fig 4C) shows similar results, with a peak around *α* = 0.4 − 0.5; although marginally weaker, the peak correlation for simulations including within-hospital delays was still over Kendall *τ* = 0.7. This indicates that a hospital’s direct connections to its neighbouring hospitals are a good enough predictor of its risk of admitting patients colonised with antibiotic-resistant bacteria from anywhere in the country. However, betweenness centrality (Fig 4D) showed far weaker correlation with the mean time to infection. This is probably a result of the dense structure of the hospital network: there exist many alternative routes next to the shortest paths through the hospitals with high betweenness that are just marginally longer and therefore – epidemiologically speaking – just as important. None of the tested centrality metrics showed clear geographical correlation (Fig 5A, 5B & 5C), while hospitals with high degree or closeness centrality were the same (mainly the large tertiary care) institutions (Fig 5D & 5E).

### Relation between network and geographical distance

**Fig 6:**
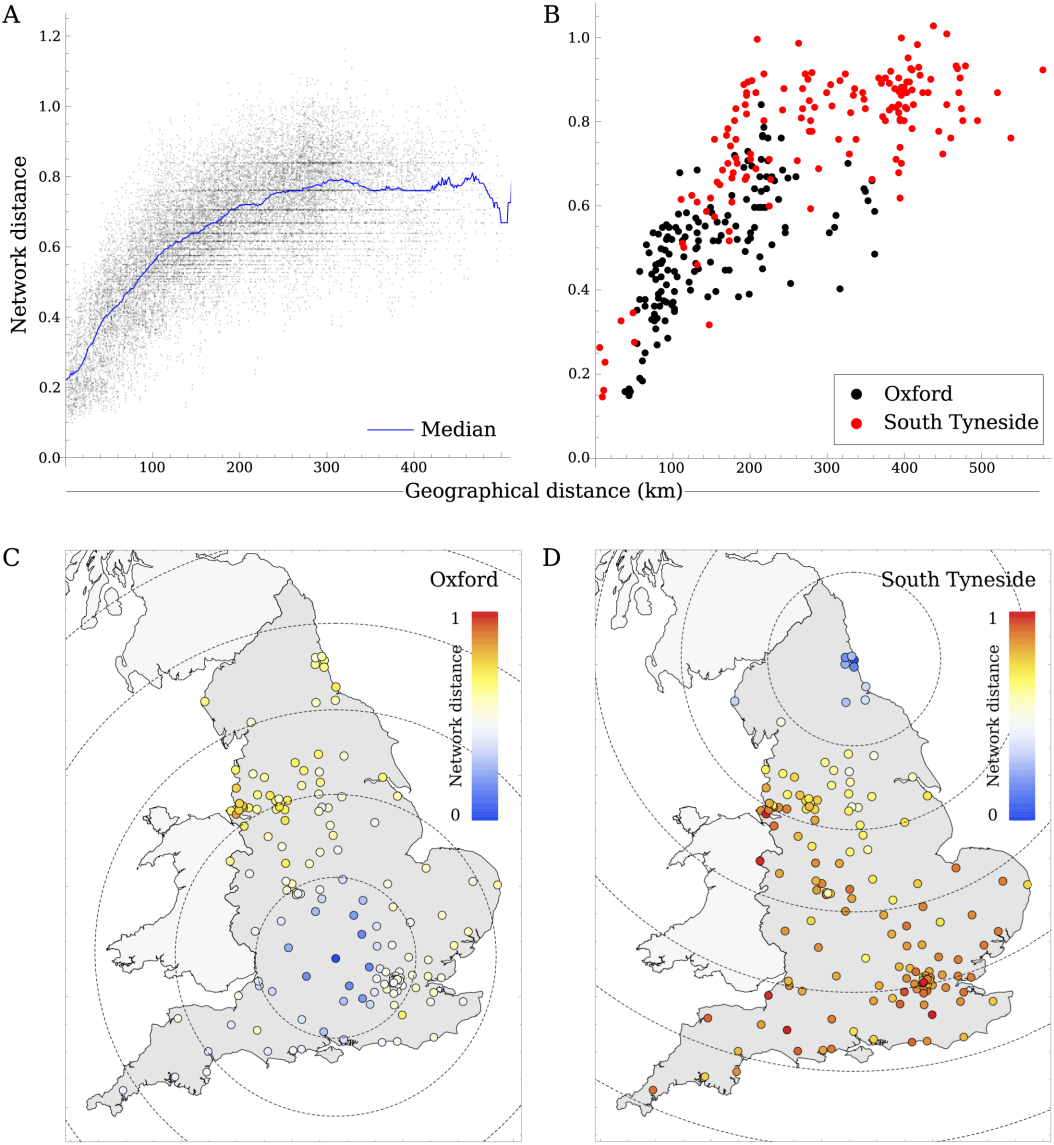
Association between network distance and geographical distance. A) The closest hospitals, within 200km, are also closest in the network, but hospitals further away are more or less all the same distance away in the network. B) The relation between geographical and network distance measured from the Oxford University Hospitals (Black) and the South Tyneside Hospital (Red) to all other hospitals. The network distance from C) Oxford and D) South Tyneside hospitals, dashed circles show geographical from the initial hospital at intervals of 100km.

Using *α* = 0.25, we observed that for hospitals in close proximity, network and geographical distance were strongly related, with hospitals sharing many patients also being geographically close to one another. However, hospitals that were geographically further away than about 200km all showed more or less the same network distance (Fig 6A). The network distance to these distant hospitals depended fairly strongly on the initial hospital they were measured from (Fig 6B, 6C and 6D).

### Removing weak links

**Fig 7:**
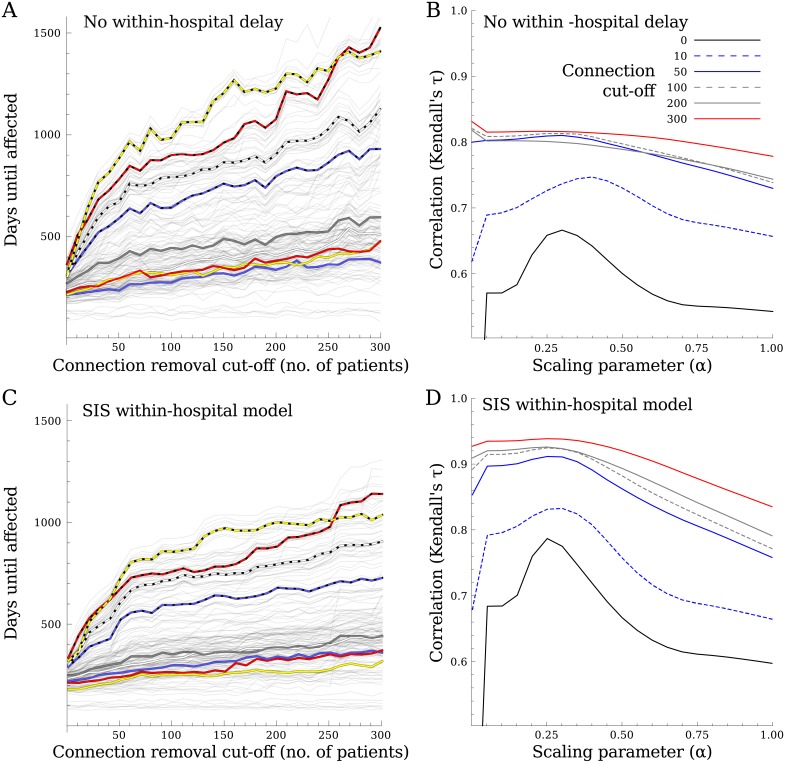
To test the effect of weak links, any connection with fewer patients than varying thresholds were removed. Thresholds with incremental steps of 10 patients were used, until the first hospital became disconnected from the rest of the network at 308 patients. A&C) The time until hospitals (thin grey lines) and referral regions (thick coloured lines) are affected as a function of the strength of the weak links being removed. B&D) The correlation between the simulated spread and the measured distance, when using networks with the weakest links removed for a number of thresholds. A&B) show the results for simulations without the within-hospital delay, C&D) show the results using the SIS within-hospital model, with a different parameter set to result in similar time to infection using all connections. Both the time to infection and the correlations increase when removing the weak links. If more of the weak links are removed, the exact value *α* starts to matter less for the correlation between time to infection and network distance.

As all network metrics favoured the number of connections or steps instead of the weight of the connections, to test their influence we removed the all weak links below thresholds of 10, 50, 100, 200, or 300 patients from the network. Using the hospital in Oxford as the initial outbreak hospital, the time to infection of other regions increased as the threshold used to remove links increased (Fig 7A & 7C), in particular for a subset of regions, where hospitals became completely disconnected from the rest of the network after removing all connections with fewer than 308 exchanged patients. Removing weak links had no effect on the dynamics within the initial region, in contrast to the spread between regions. Furthermore, the optimal estimate of *α* increased as the threshold increased, focussing the metric more on connection weight than on number of steps (Fig 7B & 7D).

## Discussion

We have shown that a hospital’s risk of importation of hospital-associated pathogens can be estimated using the hospital network, by taking both the number of patient movements and the number of intermediate hospitals in possible routes between them into account. The number of shared patients seems an appropriate distance metric for strongly connected hospitals. However, when estimating the distance between hospitals further apart in the patient referral network, it is insufficient to measure the shortest path using just the number of shared patient, as each extra step in the route confers a delay in the spread of a pathogen, and needs to be taken into account. We found that the existence of a connection between hospitals, i.e. whether or not any patients were shared between hospitals within a year, was more important than the strength of these connections. Strictly looking at directly shared patients between hospitals may underestimate the risk posed by hospitals outside the referral region.

An accurate measure of distance between hospitals is important to enable effective adjustment of IPC efforts to the current AMR situation, because these need to take the risk posed by resistance problems in other hospitals into account. An accurate risk assessment depends not only on reliable reports of the prevalence of AMR in all hospitals, but also on a correct estimation of the ‘distance’ to each hospital in terms of infection risk, because smaller problems in hospitals nearby can pose larger risks than large problems in hospitals far away. Measuring distance between hospitals through a hospital network provides one method of estimating the risk of direct or indirect transmission of a pathogen between them; however, exactly how this distance should be measured has not been investigated to date. This paper contributes to finding the best metric for measuring this distance.

The risk for individual hospitals in the absence of knowledge about the current situation in other hospitals can be estimated using their degree centrality, as well as their closeness centrality, mirroring observations made in human host-to-host networks [2, 35, 36]. Degree centrality has the advantage of being a local metric, which can be estimated by an individual hospital organisation without knowledge of the entire hospital network. It is therefore easier to apply than closeness centrality, whose use of all shortest path lengths across the network is more exact, yet would require an individual hospital to know the complete network structure. Because degree centrality can be estimated by hospitals themselves, they can either use it as an operational indicator to be lowered (by breaking away weak connections) to reduce risk of introduction, or use it to compare their position with other hospitals willing to report their centrality, with the goal of adjusting IPC efforts to their own level of risk.

The speed of spread to far-away hospitals seems to strongly depend on the presence of the many weak links in the hospital network. The removal of these weak links resulted in longer time to infection of some hospitals, in particular those further away in the network, while leading to a higher estimate of the scaling parameter *α*, i.e. incorporates more of the connections’ weight in the distance metric. This indicates that the weak links in the network can be ideal routes for hospital-associated-pathogens to travel further faster. The combined weight of all weak links, together with enough waiting time, increases the probability that one of them will result in a successful introduction far away. This means that preventing introductions along the weak links, for instance by preferentially screening and isolating patients coming from ‘unusual’ hospitals, could potentially slow the spread of hospital-associated pathogens over longer distances.

However, despite the influence of the weak links, closely neighbouring hospitals that exchange many patients are still at greatest risk of transmitting between each other [37], and are the first ones to be affected after the initial outbreak. These closely connected health care institutions, which include long-term care facilities and nursing homes, also affect each other’s prevalence of hospital-associated pathogens, depending on the length of stay of patients, the size of the institutions, the hospitals they exchange patients with, and the total number of patients they exchange [11, 38, 39]. This reiterates the need for regional coordination of infection prevention and control [26]. Focussing control efforts on the weak links may therefore be advantageous on a global level, affecting the entire network, while focussing on the stronger links makes more sense on a local level.

It is only once a pathogen has spread to the hospitals in the same region that the exact number of patients shared with the affected hospitals becomes a less important predictor of the risk of introduction, because the cumulative weight of routes involving weak links increases dramatically and the number of possible routes between affected and unaffected regions expand rapidly. In this respect, hospital networks behave as small-world networks, with the weak connections offering shorter routes for the pathogen to spread to otherwise distant hospitals. This also means that the marked difference between geographical and network distance between hospitals (which are far apart geographically) has important implications for control efforts, because a regional outbreak in, for instance, the South-West of England just as easily forms a threat to hospitals in the North-East as it would in the Midlands.

Preventing the transition from a regional outbreak to a national problem forms a formidable challenge for IPC efforts, as the possible routes between affected and unaffected regions rapidly expands. At this stage, preventing introductions through screening patients moving from other hospitals becomes very difficult, as well as costly, because many more patients have to be screened and isolated. The many different routes colonised patients are coming from makes it harder to define clear risk groups based on previous hospital admissions. Moreover, as the number of hospitals experiencing considerable infection pressure increases, the chances of more hospitals failing control also increase, creating a chain reaction culminating in all hospitals being affected. This shows the importance of controlling any AMR or healthcare-associated infection problem as soon as it is detected, before it can cause wider problems that are harder to control.

### Limitations

We considered three exemplar network metrics, capturing different structures, while more are available. It would also possible to evaluate a larger number of metrics in a meta-modelling approach to determine the full effect of the network structure on the risk of a hospital receiving hospital-associated pathogens; in particular, as effects that are not captured by one network metric may be captured by another. This could result in a combined metric, with different metrics adding to the hospital’s risk, which we deem beyond the scope of this single paper.

It should be noted that the epidemiological distance used in our simulation is purely based on the simulations using the observed hospital network. The simulation model is an approximation, and results may be influenced by specific assumptions. For example, we assume that all patients within a single hospital trust have an equal opportunity to transmit the pathogen to each other. However, the physical and organisational structure of a hospital affects these probabilities, resulting in preferential transmission between patients admitted to the same ward, often with similar conditions. Apart from clustering in the network, medical conditions may also alter the susceptibility of patients, causing different rates and different connectivity depending on the particular condition. Differences in rates may be exacerbated in wards with larger proportions of frail patients, resulting higher mortality. This could imply that the condition- or ward-specific sub-networks might even have to be weighted differently depending on the question at hand.

Sub-networks may also exist on the level of the institutions, as the network is based on hospital organisations that could comprise multiple hospital sites. However, these hospital sites within one organisation can be expected to share even more patients than hospital organisations within a single region, thus creating an extra layer within the network. Although these assumptions may alter the absolute distance between hospitals in the network, the relative difference is unlikely to change dramatically as we can expect the within-trust networks to be much more tightly connected, thus forming a single entity, compared to the between-trust network.

### Conclusion

The network distance between hospitals is not merely a reflection of the number of shared patients between them, but also the number of intermediate hospitals that need to be passed en-route from one to the other. Because the network of hospitals consists of many weak links, formed by hospitals sharing very few patients, the distance between far away hospitals may therefore easily be underestimated. This particularly applies when a pathogen has spread to other hospitals in the initial region and many more weak links become available for its onward spread to different regions. Our observations hold important implications for IPC efforts: if a local outbreak is not controlled in time, colonised patients will appear in other regions from unexpected hospitals, making import screening ever more difficult.

## Supporting information

S1 Fig Determining the best number of model iterations. After generating 1000 simulations of the model, we randomly selected a fixed number of simulations, calculated the mean times to infections, and their correlation with (A) network distance (B) closeness centrality, (C) degree centrality, and (D) betweenness centrality using *α* = 0.25. This process was repeated 25 times for each number of simulations between 10 and 250. Increasing the number of simulations increases the correlation with network distance (A) and decreases the variability in the estimated correlation with network characteristics (B) (C) (D). After 100 simulations, improvements in the estimates and variability are marginal. The correlations between (E) network distance and mean time to infection as a function of *α* for 10, 50, 100, and 200 simulations per initial, as well as (F) the correlation between closeness and mean time to infection, show no shift in the optimal value of *α* depended on the number of simulations.
(EPS)

S2 Fig Sensitivity analysis using different introduction probabilities for the model without within-hospital delay. (EPS)

S3 Fig Sensitivity analysis using different introduction probabilities for the model using the SIS within-hospital model with beta = 0.1 and delta = 0.08. (EPS)

S4 Fig Sensitivity analysis using different equilibrium prevalences (adjusting delta) for the model using the SIS within-hospital model with faster within-hospital spread, using beta = 1.0. (EPS)

S1 Table The contact matrix between all hospital trust in England, showing the number of patients received from other hospitals during financial year 2014–2015. (XLS)
